# Comparative Positioning of Orforglipron Among Selected GLP-1 Receptor Agonist Benchmarks: A Narrative Review

**DOI:** 10.7759/cureus.110178

**Published:** 2026-06-03

**Authors:** José Castro-Gamboa, Jeaustin Mora-Jiménez, Sebastián Arguedas-Chacón, Esteban Zavaleta-Monestel

**Affiliations:** 1 Pharmacy, Universidad de Costa Rica, San José, CRI; 2 Research, Hospital Clínica Bíblica, San José, CRI; 3 Pharmacy, Hospital Clínica Bíblica, San José, CRI

**Keywords:** glp-1 receptor agonist, nonpeptide agonist, obesity, oral semaglutide, orforglipron, small molecule, type 2 diabetes

## Abstract

Orforglipron is an oral, nonpeptide, small-molecule glucagon-like peptide-1 receptor agonist developed for type 2 diabetes and obesity. This narrative review evaluates its comparative positioning among marketed GLP-1 receptor agonists by integrating structural, pharmacological, clinical, and practical evidence.

A narrative literature review was conducted using PubMed/MEDLINE, Embase, Scopus, Web of Science, ClinicalTrials, official prescribing information, and citation tracking. The synthesis was organized by comparative domains, including molecular structure, receptor pharmacology, early clinical pharmacology, direct comparative evidence, contextual comparator evidence, and practical administration. From 245 screened records, 88 duplicates were removed, 157 unique records were assessed, and 35 orforglipron-focused sources were retained. Seven additional comparator trials were selected through targeted citation verification because they represented clinically relevant benchmarks for oral semaglutide in type 2 diabetes and obesity, danuglipron as another oral small-molecule GLP-1 receptor agonist, and injectable semaglutide as a high-efficacy class comparator.

Current evidence identifies several domains relevant to the comparative positioning of orforglipron, including its nonpeptide small-molecule structure, receptor pharmacology, once-daily oral dosing profile, and direct comparator evidence against dulaglutide and oral semaglutide. Food-effect and prescribing-information sources describe fewer food-related administration constraints for orforglipron than for oral semaglutide. However, indirect comparisons remain limited by differences in populations, doses, follow-up duration, comparators, and endpoints.

At present, orforglipron should be viewed as an oral small-molecule GLP-1 receptor agonist with emerging comparative evidence, rather than as a broadly superior replacement for existing agents. Further long-term comparative, cardiovascular, renal, safety, adherence, and real-world effectiveness data are needed to define its final place in therapy.

## Introduction and background

Orforglipron is an orally administered, nonpeptide, small-molecule agonist of the glucagon-like peptide-1 receptor (GLP-1R) developed as an alternative to injectable peptide-based GLP-1 receptor agonists and to the currently marketed oral peptide formulation of semaglutide [1-3]. Its relevance extends beyond oral delivery alone. The foundational structural study by Kawai and colleagues showed that LY3502970/orforglipron activates GLP-1R through a nonpeptide binding mode and behaves as a partial agonist with signaling biased toward G-protein activation over beta-arrestin recruitment, providing a mechanistic basis for its differentiation from peptide agonists [1].

The early clinical development program has also been published in peer-reviewed form. A phase Ia trial evaluated safety, tolerability, pharmacokinetics, and pharmacodynamics in healthy participants, whereas a distinct phase Ib trial assessed multiple ascending doses in adults with type 2 diabetes [4,5]. A dedicated food-effect study subsequently evaluated the relationship between prandial state and once-daily oral administration of orforglipron, providing evidence relevant to administration requirements when compared with oral semaglutide [6].

Direct comparative evidence against marketed GLP-1 receptor agonists remains limited but is no longer absent. In a randomized phase II study, orforglipron was evaluated against placebo and dulaglutide in adults with type 2 diabetes [7]. More recently, the ACHIEVE-3 phase III trial directly compared once-daily oral orforglipron with oral semaglutide, thereby providing head-to-head evidence against the only marketed oral GLP-1 receptor agonist [8]. In parallel, a recent mechanistic pharmacology paper and a comprehensive review have expanded the evidence base available for narrative synthesis [2,9].

From a clinical-practical standpoint, the current US prescribing information for Foundayo states that orforglipron may be taken once daily with or without food [3]. By contrast, the US prescribing information for Rybelsus instructs patients to take oral semaglutide at least 30 minutes before the first food, beverage, or other oral medications of the day and with no more than 4 ounces of plain water only [10]. This contrast identifies administration requirements as a relevant practical comparison domain, although the available label and food-effect evidence should not be interpreted as direct evidence of improved adherence, persistence, patient preference, or clinical outcomes [3,6,10].

Although recent reviews have summarized the overall development of orforglipron, the field still benefits from a focused narrative review built around a more clinically useful question: in which domains does orforglipron appear differentiated, potentially advantageous, broadly comparable, or still uncertain when compared with marketed GLP-1 receptor agonists [2]. Organizing the evidence around structure-function relationships, direct versus indirect comparative evidence, and practical prescribing issues may therefore offer clearer value than another general summary of efficacy and safety.

Accordingly, this narrative review aims to critically compare orforglipron with marketed GLP-1 receptor agonists by integrating structural, pharmacological, clinical, and practical evidence. Specifically, the review examines the molecular and receptor-level features that differentiate orforglipron from peptide-based GLP-1 receptor agonists, summarizes its early clinical pharmacology and oral dosing profile, synthesizes available direct comparative evidence against dulaglutide and oral semaglutide, and evaluates practical administration differences using official prescribing information. By organizing the evidence across these domains, this review seeks to clarify where orforglipron currently appears differentiated, potentially advantageous, broadly comparable, or still uncertain relative to marketed GLP-1 receptor agonists.

## Review

Materials and methods

Review Design

This manuscript has been prepared as a narrative review with a clinically oriented comparative focus. The review is not intended to perform a formal quantitative synthesis or to establish pooled estimates of treatment effect. Instead, it is designed to integrate and critically interpret the available evidence on orforglipron in relation to marketed glucagon-like peptide-1 receptor agonists (GLP-1RAs). The review framework is intended to examine how the available evidence positions orforglipron relative to currently marketed GLP-1 receptor agonists across mechanistic, clinical, and practical-administration domains, including areas of apparent distinction, comparability, and uncertainty.

Reporting of this narrative review was guided by the Scale for the Assessment of Narrative Review Articles (SANRA) [11], which was used as a methodological framework to strengthen transparency in the statement of aims, literature search description, referencing, evidence-level presentation, and endpoint reporting. Because this manuscript was designed as a narrative review rather than a systematic review or meta-analysis, Preferred Reporting Items for Systematic reviews and Meta-Analyses (PRISMA) was not used as the primary reporting framework. 

The narrative format was selected because the evidence base was heterogeneous and included structural biology studies, mechanistic pharmacology papers, early-phase pharmacology trials, active-comparator clinical studies, review articles, and official prescribing information documents. The completed synthesis was therefore organized by comparative domain rather than by publication chronology alone.

This review used a structured but non-systematic narrative approach. The structured components included database searching, duplicate removal, predefined source eligibility and relevance criteria, source-level verification, and extraction of key bibliographic, mechanistic, clinical, and practical-administration information. These steps were used to improve the transparency and reproducibility of the evidence map. However, the synthesis remained narrative and interpretive: no formal risk-of-bias tool was applied, no quantitative pooling was performed, and no claim of exhaustive systematic evidence capture or comparative treatment-effect estimation was intended. Direct comparative trials, mechanistic studies, regulatory documents, and selected contextual comparator trials were interpreted according to their relevance to the predefined comparative domains rather than combined into pooled estimates.

Information Sources

The primary electronic databases selected for this review were PubMed/MEDLINE, Embase, Scopus, and Web of Science. These databases were chosen to ensure broad coverage of biomedical, pharmacological, and translational literature relevant to orforglipron and its comparison with marketed GLP-1 receptor agonists.

To complement database searching, additional sources included ClinicalTrials, official US Food and Drug Administration (FDA) prescribing information, DailyMed, and backward and forward citation tracking from key seed articles and recent review papers. Regulatory documents were included because they provide clinically relevant information on dosing instructions, administration constraints, and practical prescribing considerations that are not always fully addressed in journal publications.

Search Strategy

The search strategy was adapted to each database according to its syntax, indexing system, and retrieval scope. Broad initial searches were performed to maximize sensitivity, after which filters for publication year, language, study type, and human population were applied depending on the review domain. Structural and mechanistic searches were intentionally left without a human-only restriction to preserve preclinical and translational evidence relevant to molecular differentiation, whereas clinical-comparative searches were restricted to human studies whenever appropriate. Regulatory sources were searched separately and used only for official administration and prescribing claims. To preserve methodological transparency while maintaining readability in the main manuscript, the complete database-specific search strategies, recommended filters, and methodological notes are provided in the Appendix. The completed synthesis was organized by comparative domain rather than by publication chronology alone.

Regulatory Document Search

Regulatory sources were searched separately from bibliographic databases. Official US prescribing information available through FDA labeling records and/or DailyMed was used exclusively to support claims related to dosing instructions, food requirements, dose escalation, and practical prescribing considerations. These sources were not treated as primary evidence of comparative efficacy. For citation consistency, official label documents cited in the reference list were normalized as US Food and Drug Administration prescribing-information records, irrespective of whether the source was retrieved through FDA labeling records or DailyMed.

Eligibility Criteria

Eligible sources included: 1) preclinical studies focused on receptor binding, structural biology, signaling behavior, or medicinal chemistry of orforglipron/LY3502970; 2) phase I to phase III clinical trials evaluating orforglipron; 3) studies including an active comparator relevant to marketed GLP-1 receptor agonists; 4) narrative reviews, systematic reviews, meta-analyses, and other evidence syntheses relevant to comparative interpretation; and 5) official regulatory or prescribing-information documents used to compare dosing instructions, use constraints, and practical prescribing implications.

Exclusion Criteria

Excluded sources comprised: (1) non-scholarly promotional material used as primary evidence; (2) conference abstracts without sufficient methods or results for interpretation; (3) duplicate reports superseded by a full publication; and (4) articles that merely repeated class-level efficacy data without contributing to the comparative question of the review.

Duplicate Handling

Before narrative screening, the RIS file was reviewed to identify duplicate records and repeated reports of the same source. Duplicates were identified primarily by DOI and, when DOI information was unavailable, by normalized title matching. This process was used to avoid double-counting of the same publication within the evidence map. From 245 imported records, 88 duplicates were removed, leaving 157 unique records for narrative relevance screening.

Source Selection and Narrative Relevance Screening

Study selection was conducted in a staged manner using the screened RIS file and the predefined eligibility criteria. The complete search framework used to generate and organize the screened records is provided in the Appendix. After duplicate removal, 157 unique records were screened. Thirty-five records from the RIS file were retained for the orforglipron-focused synthesis, including official prescribing-information records. The remaining 122 unique records were not retained because they were duplicate-independent but insufficiently relevant, registry-only or non-article records not used as citable primary evidence, or broader GLP-1/obesity/diabetes records that did not contribute directly to the comparative question.

To preserve the focus of the review, seven external comparator trials were added separately by targeted citation verification for contextual discussion only: PIONEER 4, PIONEER 3, OASIS 1, oral semaglutide 25 mg, danuglipron in type 2 diabetes, danuglipron in obesity, and STEP 1. These comparator trials were not counted as orforglipron search hits; they were selected because they represented clinically relevant comparator categories for the review question: marketed oral semaglutide in type 2 diabetes, higher-dose oral semaglutide in obesity, another oral small-molecule GLP-1 receptor agonist, and injectable semaglutide as an efficacy benchmark.

At title, abstract, and metadata screening, exclusion was documented at the level of broad reason: 70 broad GLP-1/obesity/diabetes records that were not specific enough for the orforglipron-focused comparative question; 42 trial-registry or non-article records not retained as citable primary evidence; seven records not relevant to the predefined review question; and three potentially relevant small-molecule/GLP-1 records not retained after relevance appraisal because more directly relevant orforglipron-specific evidence was available.

Full-Text Review Procedure

Full-text and source-level review was performed to verify study design, confirm direct relevance to orforglipron/LY3502970, determine whether comparative claims were based on direct or indirect evidence, and assess whether each source contributed meaningfully to one or more predefined synthesis domains. Sources that did not meet these conditions were not retained in the final narrative synthesis.

Data Extraction

For each included source, the following information was extracted when applicable: author and year of publication, journal or source type, study design, population or experimental model, comparator, main structural or mechanistic finding, principal pharmacological finding, relevant clinical outcomes, administration-related observations, major limitations, and relevance to the review question.

For clinical studies, particular attention was paid to glycemic outcomes, bodyweight effects, tolerability, discontinuation patterns, active comparators, and practical implications of oral dosing. For mechanistic and structural studies, emphasis was placed on receptor binding mode, signaling bias, nonpeptide agonism, and how these features differentiate orforglipron from peptide-based GLP-1 receptor agonists. Regulatory documents were used specifically for dosing instructions, food-related administration requirements, and other clinically relevant prescribing considerations.

Quality and Relevance Appraisal

Because this work was designed as a narrative review rather than a formal systematic review, no standardized risk-of-bias instrument was applied. Instead, a structured source-level relevance and quality appraisal framework was used to determine how each source contributed to the narrative synthesis. This framework considered six dimensions: 1) evidentiary role, including whether the source was mechanistic, clinical, comparative, secondary, regulatory, or contextual; 2) directness to orforglipron/LY3502970; 3) relevance to the predefined comparative domains; 4) methodological maturity, including whether evidence was preclinical, phase I, phase II, phase III, review-level, or regulatory; 5) comparative strength, including whether comparisons were direct, indirect, active-comparator, placebo-controlled, or contextual; and 6) interpretive limitations, including study duration, population specificity, comparator coverage, tolerability assessment, and generalizability.

Bibliographic verification included confirmation of authorship, title, journal, year, volume, issue, pagination, and DOI when applicable. References were also checked to ensure that they addressed orforglipron specifically or, for targeted comparator trials, contributed directly to the contextual benchmarking of oral semaglutide, danuglipron, or injectable semaglutide.

Quality and relevance appraisal was conducted pragmatically according to the nature of the source. Priority was given to peer-reviewed structural studies, mechanistic pharmacology papers, phase I-III clinical trials, active-comparator studies, and official regulatory documents. Secondary literature, including narrative reviews and broader evidence syntheses, was used primarily for contextualization and interpretive support rather than as a substitute for primary evidence.

Included references were categorized according to their evidentiary role in the review: 1) foundational evidence, such as structural biology and mechanistic pharmacology studies; 2) primary clinical evidence, including phase I, phase II, and phase III trials; 3) comparative evidence, whether direct or indirect; 4) secondary interpretive evidence, including reviews and evidence syntheses; 5) regulatory/practical evidence, such as official prescribing information; and (6) targeted external comparator evidence used for contextual benchmarking.

In addition, limitations of each reference were taken into account during interpretation, including whether the evidence was preclinical or clinical, direct or indirect, exploratory or confirmatory, early-phase or phase III, blinded or open-label, and whether comparator coverage across the GLP-1 receptor agonist class was broad or limited.

Narrative Synthesis Approach

The final synthesis was organized by predefined comparative domains rather than by publication chronology alone. This domain-based approach was chosen to better align the evidence with the clinical question of whether the available evidence supports areas of distinction, comparability, or uncertainty for orforglipron relative to marketed GLP-1 receptor agonists.

The synthesis was structured around the following domains: 1) molecular and structural differentiation, including the nonpeptide small-molecule basis of orforglipron and its receptor binding mode; 2) receptor pharmacology, including signaling bias and mechanistic pharmacology; 3) early clinical pharmacology, including phase Ia, phase Ib, food-effect, disposition, and bioavailability evidence; 4) direct comparative clinical evidence, particularly comparisons with dulaglutide and oral semaglutide; 5) indirect and contextual comparative evidence, including reviews and broader interpretive literature; 6) administration and practical clinical relevance, including the contrast between unrestricted orforglipron dosing and the fasting-related requirements of oral semaglutide; and 7) targeted external comparator evidence used to benchmark interpretation without expanding claims beyond available data.

Results

Study Selection and Overall Characteristics

The screening file contained 245 records. Deduplication by DOI and normalized title removed 88 records, leaving 157 unique records for title, abstract, and metadata screening. Of these, 35 RIS-derived records were retained for the orforglipron-focused synthesis. The excluded 122 unique records comprised 70 broad GLP-1/obesity/diabetes records that were not specific enough for the review question, 42 trial-registry or non-article records not retained as citable primary evidence, seven records outside the predefined question, and three small-molecule/GLP-1 records not retained after relevance appraisal because more directly relevant orforglipron-specific evidence was available.

The 35 orforglipron-focused evidence sources comprised two official prescribing documents [3,10], two foundational mechanistic or structural primary studies [1,9], five early clinical pharmacology or disposition studies [4-6,12,13], eight primary clinical efficacy, biomarker, or comparator-focused studies [7,8,14-19], and 18 review-level or evidence-synthesis sources [2,20-36].

Structural and Molecular Differentiation

The structural evidence identified orforglipron as a distinct nonpeptide, small-molecule GLP-1 receptor agonist rather than an oral reformulation of an existing peptide GLP-1 receptor agonist [1,9]. The foundational structural study of LY3502970/orforglipron and the later pharmacological work supported a mechanistic distinction based on nonpeptide receptor-binding behavior and receptor activation profile [1,9]. This evidence supports the evaluation of orforglipron as mechanistically distinct from peptide-based GLP-1 receptor agonists, but it does not by itself establish a separate pharmacologic class or clinical superiority over peptide GLP-1 receptor agonists.

The surrounding review literature reinforced this mechanistic distinction by positioning small-molecule oral GLP-1 receptor agonists as a separate development strategy with potential implications for oral delivery, manufacturability, and dosing practicality [2,20,23,29,33,36]. These sources were used as interpretive context rather than as substitutes for the primary structural and pharmacological studies.

Receptor Pharmacology

The receptor-pharmacology domain was anchored by the structural and mechanistic studies and supported by broader mechanistic reviews [1,9,36]. The key finding for this review was not only that orforglipron activates GLP-1R, but that it does so through a nonpeptide agonist mechanism that differs from peptide agonism. This pharmacological profile supported the rationale for comparative clinical development, while also requiring caution: mechanistic differentiation should be interpreted as biological plausibility and therapeutic rationale, not as proof of broader clinical dominance across the GLP-1 receptor agonist class.

Early Clinical Pharmacology

The early clinical pharmacology evidence showed that once-daily oral dosing was feasible across healthy volunteers and type 2 diabetes populations [4,5]. In the phase Ia study, 92 healthy participants received single or multiple ascending doses; pharmacokinetics were approximately dose proportional, the reported half-life supported once-daily dosing, and gastrointestinal events were the most common adverse events [4]. In the phase Ib study in type 2 diabetes, 51 participants received orforglipron and 17 received placebo; at week 12, mean HbA1c changes across orforglipron doses ranged from -1.5% to -1.8%, compared with -0.4% with placebo, and bodyweight changes ranged from -0.24 to -5.8 kg, compared with a 0.5 kg increase with placebo [5].

The food-effect study was relevant to the practical-administration comparison because it evaluated the relationship between food intake and orforglipron pharmacokinetics, safety, and tolerability under study conditions [6]. Additional phase I evidence expanded characterization of disposition, absolute bioavailability, and pharmacodynamic response [12,13]. The disposition study reported a mean absolute oral bioavailability of 79.1% and predominantly fecal recovery of administered radioactivity, while the Japanese phase I study showed glycemic and bodyweight reductions over 12 weeks with a tolerability pattern consistent with other GLP-1 receptor agonists [12,13]. Together, these studies connected the molecule's oral small-molecule design with a clinically practical dosing profile.

Direct Comparative Clinical Evidence

The phase II type 2 diabetes trial provided the first active-comparator framework in the included primary evidence by comparing once-daily oral orforglipron with placebo and once-weekly dulaglutide [7]. In this 26-week trial, 383 participants were enrolled; under the efficacy estimand reported by the trial, mean HbA1c reduction with orforglipron reached up to -2.10%, compared with -0.43% with placebo and -1.10% with dulaglutide, and mean bodyweight change reached up to -10.1 kg versus -2.2 kg with placebo and -3.9 kg with dulaglutide [7]. These values should be interpreted within the trial's prespecified estimand framework, dose-escalation design, and follow-up duration. Gastrointestinal events were the most common adverse events, which aligned with the GLP-1 receptor agonist class profile [7].

ACHIEVE-3 strengthened the comparative evidence by directly comparing once-daily oral orforglipron with oral semaglutide in adults with type 2 diabetes inadequately controlled with metformin [8]. The trial randomized 1698 participants to orforglipron 12 mg or 36 mg, or oral semaglutide 7 mg or 14 mg, for 52 weeks [8]. For the treatment regimen estimation, mean HbA1c changes at week 52 were -1.71% with orforglipron 12 mg, -1.91% with orforglipron 36 mg, -1.23% with semaglutide 7 mg, and -1.47% with semaglutide 14 mg [8]. Estimated treatment differences were -0.48 percentage points for orforglipron 12 mg versus semaglutide 7 mg (95% CI, -0.65 to -0.31; p<0.0001), -0.44 percentage points for orforglipron 36 mg versus semaglutide 14 mg (95% CI, -0.62 to -0.26; p<0.0001), -0.24 percentage points for orforglipron 12 mg versus semaglutide 14 mg (95% CI, -0.41 to -0.07; p=0.0050), and -0.68 percentage points for orforglipron 36 mg versus semaglutide 7 mg (95% CI, -0.85 to -0.50; p<0.0001) [8]. Thus, the primary non-inferiority objective was met, and superiority for HbA1c reduction was shown under the trial's prespecified testing framework; however, gastrointestinal events, discontinuations due to adverse events, and mean pulse-rate increases were higher with orforglipron than with oral semaglutide [8].

Clinical Efficacy and Safety in Obesity and Type 2 Diabetes

Beyond the early and active-comparator trials, later primary clinical evidence extended the orforglipron data set into obesity, early type 2 diabetes, and obesity with type 2 diabetes [16-19]. The phase II obesity trial showed clinically meaningful weight reduction in adults with obesity or overweight, including a substantially higher proportion of participants achieving at least 10% weight loss by week 36 with orforglipron than with placebo [16]. Subsequent phase III publications expanded the evidence base to longer obesity treatment, early type 2 diabetes, and obesity in people with type 2 diabetes [17-19].

These later trials are important for interpretation because they shift the evidence base beyond proof-of-concept pharmacology. They demonstrate that orforglipron has been evaluated in clinically relevant metabolic-disease populations in which weight reduction, glycemic control, gastrointestinal tolerability, discontinuation, and cardiometabolic markers must be interpreted together [16-19]. However, they do not remove the need for longer-term outcomes evidence, especially for cardiovascular, renal, adherence, and post-approval effectiveness endpoints.

Indirect Comparative Evidence

The retained secondary literature consisted of narrative reviews, systematic reviews, meta-analyses, network meta-analyses, and scoping reviews [2,20-36]. These sources were useful for mapping the broader oral GLP-1 receptor agonist landscape, consolidating dose-response and tolerability signals, and contextualizing orforglipron among emerging small-molecule GLP-1 receptor agonists. The review-level literature generally supported clinically meaningful effects on body weight and glycemic outcomes, but its interpretive value remained secondary to primary trials because many comparisons were indirect.

Across the indirect-comparison sources, the most consistent limitation was heterogeneity. Included studies differed in population, diabetes status, background therapy, baseline body weight or HbA1c, duration of follow-up, dose-escalation schedule, comparator choice, and estimand. For this reason, network-based or pooled findings were treated as contextual evidence and were not used to infer global superiority of orforglipron over all marketed GLP-1 receptor agonists.

Practical Administration and Clinical Relevance

A distinct practical-administration difference emerged from the food-effect study and official prescribing information [3,6,10]. Orforglipron is described as an oral small-molecule GLP-1 receptor agonist that can be taken once daily with or without food, whereas marketed oral semaglutide has fasting-related administration requirements [3,6,10]. This difference is relevant to the practical comparison of oral GLP-1 receptor agonists, but the available food-effect and prescribing-information evidence should not be interpreted as direct evidence of improved adherence, persistence, patient preference, or clinical outcomes.

The practical implication should therefore be interpreted as a difference in administration requirements, not as independent proof of greater clinical effectiveness or real-world adherence. The strongest current basis for this practical distinction is the convergence of mechanistic small-molecule design, food-effect evidence, and official administration instructions [1,3,6,9,10].

Summary of the Included Evidence

**Figure 1:**
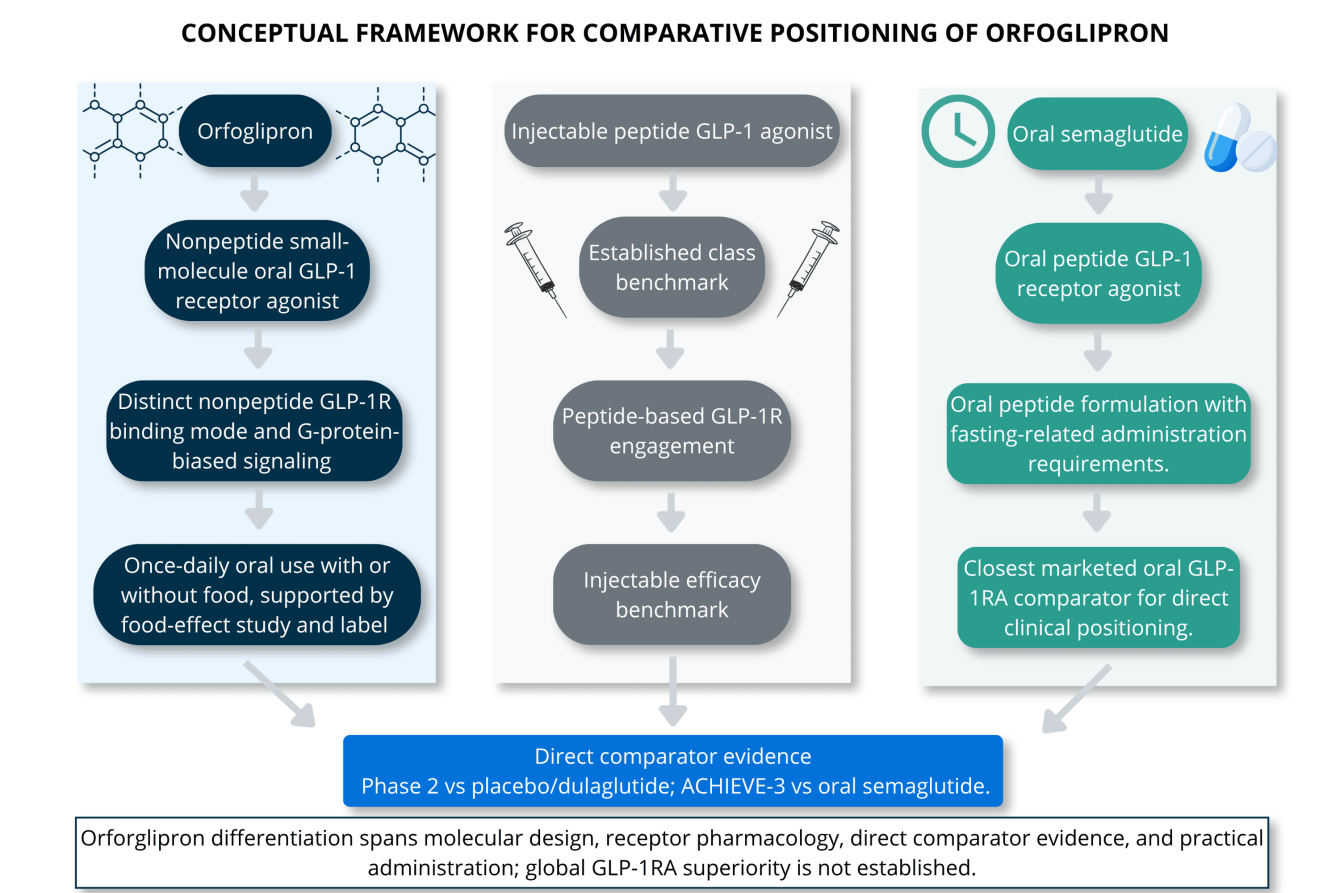
Conceptual framework for comparative positioning of orforglipron among GLP-1 receptor agonists This figure provides a conceptual synthesis of the evidence domains used to position orforglipron relative to injectable peptide GLP-1 receptor agonists and oral semaglutide. Orforglipron is shown as an oral, nonpeptide, small-molecule GLP-1 receptor agonist with a distinct receptor-pharmacology profile, fewer food-related administration constraints than oral semaglutide, and direct comparator evidence against dulaglutide and oral semaglutide. Injectable peptide GLP-1 receptor agonists and oral semaglutide are included as contextual benchmarks. The figure is intended as a visual framework for evidence interpretation and should not be read as evidence of global class-wide superiority, improved adherence, persistence, patient preference, or clinical outcomes beyond those reported in the cited studies [1,3,6–10,41]. Source: The figure was manually designed by the authors using Canva (Canva Pty Ltd., Sydney, Australia)

Overall, the literature included supported a layered interpretation of differentiation. The strongest evidence for structural and mechanistic distinction came from the foundational structural and pharmacological studies [1,9]. The strongest evidence for practical-administration differences came from the food-effect study and official prescribing information, which together defined the administration contrast with oral semaglutide [3,6,10]. The strongest direct comparative clinical evidence came from the phase II study against placebo and dulaglutide and from the phase III ACHIEVE-3 trial against oral semaglutide [7,8]. By contrast, the largest volume of literature consisted of reviews and evidence syntheses, which were essential for contextualization but did not fully resolve the question of superiority across the marketed GLP-1 receptor agonist class [2,12,13,15,20-36]. Taken together, the included evidence supports a multidomain interpretation of orforglipron's comparative positioning. Mechanistic studies define its nonpeptide small-molecule basis and receptor pharmacology, clinical trials provide direct comparator evidence against dulaglutide and oral semaglutide, and food-effect plus prescribing-information sources support practical-administration differences related to food-related requirements. These relationships are summarized in Figure 1.

Discussion

The present narrative review supports a cautious comparative interpretation of orforglipron within the GLP-1 receptor agonist landscape. The strongest basis for its current positioning is not a single endpoint in isolation, but the convergence of three evidence domains: nonpeptide small-molecule design and receptor pharmacology, feasibility of once-daily oral administration, and direct comparative evidence under specific trial conditions [1,3,6-10]. This distinction is important because oral administration and mechanistic differences do not by themselves establish broad clinical superiority. Rather, the available evidence supports orforglipron as an oral small-molecule GLP-1 receptor agonist with a distinct mechanistic and practical profile, while its comparative clinical value remains dependent on the durability of efficacy, tolerability, safety, adherence, and outcomes evidence across broader populations and longer follow-up.

**Figure 2:**
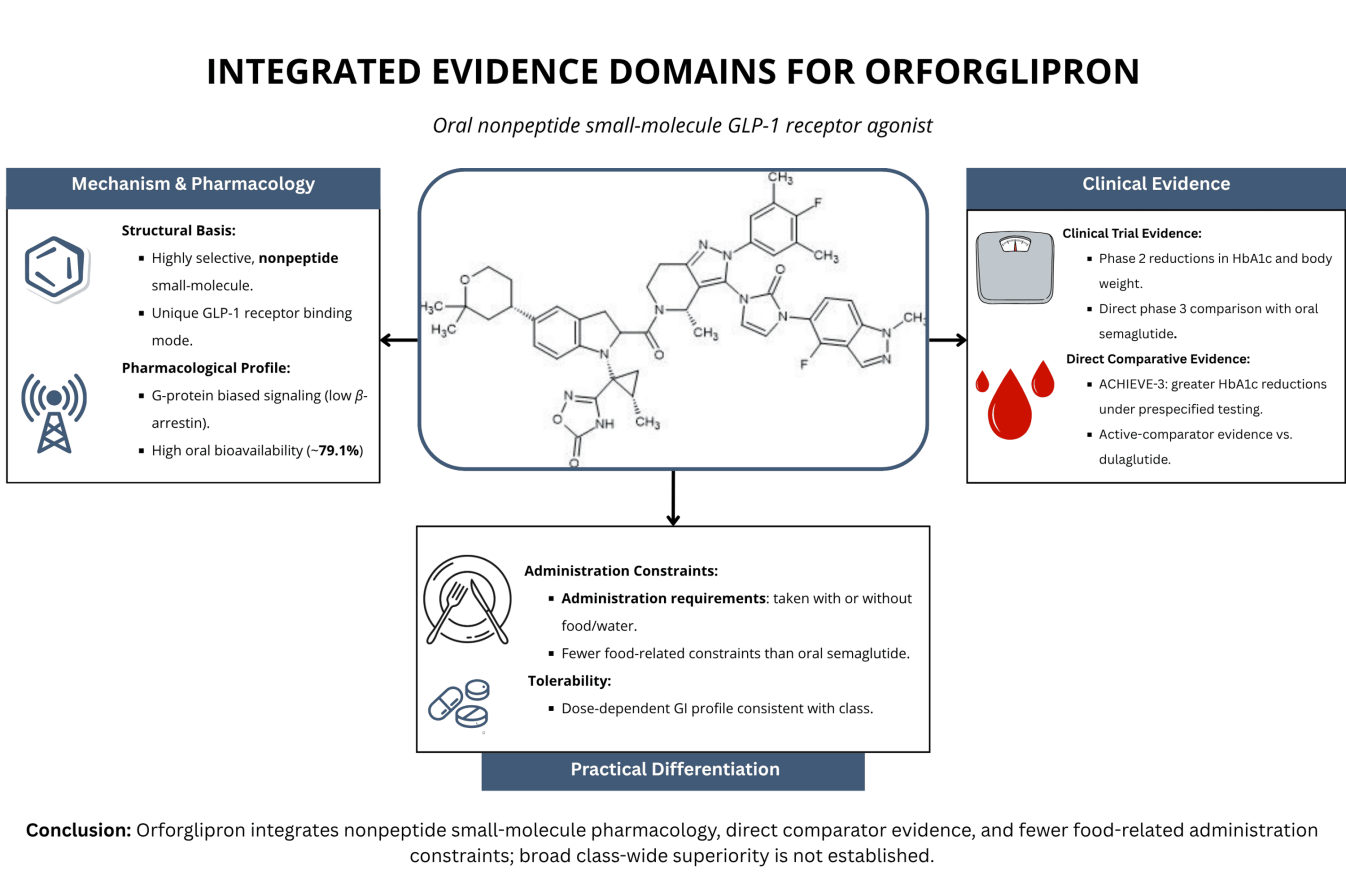
Integrated multidomain evidence supporting the comparative positioning of orforglipron This figure provides a conceptual synthesis of the mechanistic, clinical, and practical-administration evidence used to position orforglipron within the GLP-1 receptor agonist landscape. Mechanistic claims are based on structural and receptor-pharmacology studies, clinical claims are based on phase II and phase III trial evidence, and administration-related claims are based on food-effect and prescribing-information sources. The figure is intended as an interpretive framework rather than a quantitative comparison of treatment effects. It should not be interpreted as evidence of global superiority across all marketed GLP-1 receptor agonists or as evidence of improved adherence, persistence, patient preference, or clinical outcomes beyond those reported in the cited studies [1,3–10,12,16–19]. Source: The figure was manually designed by the authors using Canva (Canva Pty Ltd., Sydney, Australia).

The comparison with oral semaglutide is central because semaglutide has provided the main clinical precedent for oral GLP-1 receptor agonism. In type 2 diabetes, PIONEER 4 showed that oral semaglutide was non-inferior to subcutaneous liraglutide and superior to placebo for HbA1c reduction, while also producing greater bodyweight reduction than both liraglutide and placebo at week 26 [37]. PIONEER 3 further showed that oral semaglutide 7 mg and 14 mg produced greater HbA1c reductions than sitagliptin over 26 weeks in adults with type 2 diabetes inadequately controlled with metformin, with or without sulfonylurea [38]. These findings established oral semaglutide as an effective oral GLP-1 receptor agonist in diabetes, but they also provide the appropriate context for interpreting orforglipron: the relevant question is not simply whether an oral GLP-1 receptor agonist can work, but how a small-molecule oral agonist compares with established oral GLP-1 receptor agonist therapy across efficacy, tolerability, and administration requirements. The integrated evidence domains supporting the current interpretation of orforglipron are summarized conceptually in Figure 2. The figure is intended to organize mechanistic, clinical, and practical-administration evidence rather than provide a quantitative comparison of treatment effects.

This multidomain framework provides the basis for interpreting orforglipron against several clinically relevant, but non-interchangeable, benchmarks. Oral semaglutide evidence in type 2 diabetes, including PIONEER 3 and PIONEER 4, provides the most relevant background for interpreting ACHIEVE-3 because these studies evaluated oral semaglutide within diabetes-focused populations and dosing contexts [37,38]. Within this type 2 diabetes framework, ACHIEVE-3 is especially important because it directly compared once-daily oral orforglipron with marketed-dose oral semaglutide in adults with type 2 diabetes inadequately controlled with metformin [8]. This head-to-head design strengthens the comparative foundation of the present review beyond indirect inference alone. However, interpretation should remain bounded by the trial's design, comparator doses, population, duration, estimand framework, and safety findings. By contrast, high-dose oral semaglutide studies in obesity are discussed separately as obesity-focused weight-loss benchmarks and should not be treated as directly interchangeable with the oral semaglutide doses or diabetes-focused populations evaluated in ACHIEVE-3, PIONEER 3, or PIONEER 4.

The obesity literature provides a second important comparator framework. OASIS 1 showed that oral semaglutide 50 mg once daily produced a superior and clinically meaningful reduction in body weight versus placebo in adults with overweight or obesity without type 2 diabetes [39]. A later phase III study of oral semaglutide 25 mg also reported greater mean bodyweight reduction than placebo in adults with overweight or obesity [40]. These studies show that oral peptide-based GLP-1 therapy can produce clinically meaningful weight loss when higher obesity-focused doses are used. Therefore, the convenience of orforglipron as an oral small molecule should not be interpreted as automatically equivalent to the highest efficacy observed across the GLP-1 receptor agonist class. Instead, obesity trials of oral semaglutide define an important benchmark, but the magnitude, durability, and tolerability of orforglipron should be interpreted as separate questions from adherence, persistence, and real-world effectiveness, which remain to be established [16,17,39,40].

STEP 1 remains an essential class benchmark because it demonstrated sustained and clinically relevant weight reduction with once-weekly subcutaneous semaglutide 2.4 mg plus lifestyle intervention in overweight or obese adults [41]. This comparison is useful precisely because it is not an oral-versus-oral comparison. It highlights that route of administration and magnitude of effect are separate dimensions of therapeutic value. Orforglipron may offer fewer food-related administration constraints as an oral nonpeptide small-molecule agonist, but the benchmark established by injectable semaglutide underscores the need to avoid framing oral convenience as a substitute for direct evidence of equal or greater efficacy across the full class [16,17,41].

The emerging danuglipron literature further clarifies the broader small-molecule GLP-1 receptor agonist context. In adults with type 2 diabetes, danuglipron reduced HbA1c, fasting plasma glucose, and body weight compared with placebo, with a tolerability profile broadly consistent with GLP-1 receptor agonism [42]. In adults with obesity, danuglipron also produced statistically significant and clinically meaningful bodyweight reductions versus placebo in a dose-ranging phase IIb study [43]. These findings support the biological and clinical plausibility of orally administered small-molecule GLP-1 receptor agonists as a drug class. At the same time, reports of gastrointestinal adverse events and treatment discontinuation in the danuglipron program emphasize that small-molecule oral delivery does not eliminate the need for careful tolerability assessment [42,43]. For orforglipron, this means that the most defensible comparative claim is not that all small-molecule agonists are interchangeable, but that the currently included evidence provides a more developed orforglipron clinical data set, including direct comparator trials, than is available for contextual interpretation of danuglipron within this review [7,8,16-19,42,43].

Taken together, these external comparators refine the interpretation of the present evidence base. PIONEER 3 and PIONEER 4 demonstrate that oral semaglutide is a clinically effective oral GLP-1 receptor agonist in type 2 diabetes [37,38]. OASIS 1 and the oral semaglutide 25 mg obesity trial show that oral GLP-1 therapy can also achieve meaningful weight reduction in obesity-focused populations [39,40]. STEP 1 anchors expectations for high-efficacy injectable GLP-1 therapy [41]. Danuglipron shows that oral small-molecule GLP-1 receptor agonism is clinically feasible but also subject to tolerability and discontinuation challenges [42,43]. Against this background, orforglipron is best characterized as a distinct oral small-molecule GLP-1 receptor agonist with emerging direct and contextual evidence, rather than as a therapy whose overall superiority across the class has already been established.

Several limitations should be acknowledged. First, this is a narrative review, and the evidence base remains heterogeneous across mechanism studies, phase I trials, phase II and phase III trials, regulatory documents, and secondary syntheses. Second, many clinically relevant comparisons remain indirect because trials differ in population, baseline glycemic status, body mass index, background therapy, dose escalation, duration, estimand, and endpoint hierarchy. Third, tolerability comparisons are especially vulnerable to differences in titration schedules, ascertainment, and discontinuation rules. Finally, the absence of mature cardiovascular, renal, long-term safety, adherence, persistence, and real-world effectiveness data remains central to the clinical positioning of orforglipron. These outcomes are especially important because the ultimate value of an oral small-molecule GLP-1 receptor agonist will depend not only on glycemic and weight effects observed in clinical trials, but also on whether these benefits are durable, tolerated, and sustained in routine practice. At present, available data support a distinct mechanistic, clinical-trial, and administration profile, but they do not yet define whether orforglipron will improve long-term cardiometabolic outcomes, persistence, or real-world treatment effectiveness compared with established GLP-1 receptor agonists. These limitations do not diminish the importance of orforglipron, but they define the evidentiary boundary within which current claims should be made.

The clinical implication is therefore balanced. Orforglipron has a distinct molecular, receptor-pharmacology, and administration profile under the currently available evidence, and it now has direct comparator data against oral semaglutide [1,3,6,8-10]. Nevertheless, the broader GLP-1 receptor agonist field includes highly effective peptide-based oral and injectable agents [37-41]. The most accurate current positioning is that orforglipron could contribute to the oral GLP-1 receptor agonist landscape by combining oral delivery with small-molecule pharmacology, while its ultimate comparative value will depend on confirmation of durable efficacy, tolerability, adherence, cardiometabolic outcomes, and long-term safety in post-approval and real-world settings.

## Conclusions

Orforglipron represents a distinct oral, nonpeptide, small-molecule GLP-1 receptor agonist whose differentiation from marketed GLP-1 receptor agonists is supported by structural, pharmacological, clinical, and practical evidence. The current evidence most strongly supports differentiation in mechanism and administration, with emerging clinical support from active-comparator trials.

External comparator studies with oral semaglutide, danuglipron, and injectable semaglutide strengthen the interpretive framework of this review. They show that oral GLP-1 receptor agonism is clinically viable, that small-molecule agonism is a plausible therapeutic strategy, and that injectable semaglutide remains an important efficacy benchmark. However, these comparisons also reinforce the need for caution: orforglipron should not be described as globally superior to all marketed GLP-1 receptor agonists without broader direct comparative and long-term outcomes evidence.

Overall, orforglipron is best positioned as a promising and differentiated oral GLP-1 receptor agonist with potential practical advantages, rather than as a fully established replacement for existing oral or injectable GLP-1 receptor agonists. Further head-to-head trials, cardiovascular and renal outcomes data, long-term safety assessment, and real-world adherence studies will be essential to define its final place in therapy.

## Appendices

**Table 1:** Appendix 1 - database-specific search strategy

Database / Source	Review domain	Purpose within the review	Search strategy	Recommended filters / limits	Methodological note
PubMed/MEDLINE	Structural biology and receptor pharmacology	To identify studies describing the molecular basis of orforglipron, its receptor-binding mode, nonpeptide agonism, and signaling profile	("orforglipron"[Title/Abstract] OR "LY3502970"[Title/Abstract] OR "Foundayo"[Title/Abstract]) AND ("GLP-1 receptor"[Title/Abstract] OR "glucagon-like peptide-1 receptor"[Title/Abstract] OR GLP1R[Title/Abstract]) AND ("structure"[Title/Abstract] OR "structural basis"[Title/Abstract] OR "binding mode"[Title/Abstract] OR "small molecule"[Title/Abstract] OR nonpeptide[Title/Abstract] OR "biased agonism"[Title/Abstract] OR "G protein-biased"[Title/Abstract] OR "medicinal chemistry"[Title/Abstract] OR "receptor pharmacology"[Title/Abstract])	Publication date: January 1, 2020 to April 30, 2026; Language: English; no Humans filter; no initial article-type restriction	Structural and mechanistic searches were kept broad to avoid excluding preclinical or translational evidence relevant to molecular differentiation
PubMed/MEDLINE	Comparative clinical evidence	To identify direct and indirect comparative studies between orforglipron and marketed GLP-1 receptor agonists	("orforglipron"[Title/Abstract] OR "LY3502970"[Title/Abstract] OR "Foundayo"[Title/Abstract]) AND ("semaglutide"[Title/Abstract] OR "oral semaglutide"[Title/Abstract] OR "dulaglutide"[Title/Abstract] OR "liraglutide"[Title/Abstract] OR "exenatide"[Title/Abstract] OR "lixisenatide"[Title/Abstract] OR "tirzepatide"[Title/Abstract]) AND ("versus"[Title/Abstract] OR comparison[Title/Abstract] OR comparative[Title/Abstract] OR "head-to-head"[Title/Abstract] OR superior[Title/Abstract] OR "non-inferiority"[Title/Abstract] OR "indirect comparison"[Title/Abstract] OR "network meta-analysis"[Title/Abstract])	Publication date: January 1, 2020 to April 30, 2026; Language: English; Species: Humans; Article types: Clinical Trial, Randomized Controlled Trial, Comparative Study; Reviews retained only if relevant to indirect comparison	This search supports the distinction between direct comparative evidence and indirect interpretive evidence
PubMed/MEDLINE	Administration, convenience, and oral dosing profile	To retrieve studies relevant to food effect, fasting requirements, oral administration, convenience, gastric emptying, and practical prescribing issues	("orforglipron"[Title/Abstract] OR "Foundayo"[Title/Abstract]) AND ("oral administration"[Title/Abstract] OR food[Title/Abstract] OR fasting[Title/Abstract] OR "dosing restrictions"[Title/Abstract] OR convenience[Title/Abstract] OR "gastric emptying"[Title/Abstract] OR "drug interactions"[Title/Abstract] OR "oral dosing"[Title/Abstract])	Publication date: January 1, 2020 to April 30, 2026; Language: English; Species: Humans; Article types: Clinical Trial, Journal Article	Used to identify clinically relevant practical issues related to oral administration
PubMed/MEDLINE	Secondary literature	To identify narrative reviews, systematic reviews, meta-analyses, and broader evidence syntheses relevant to contextual interpretation	("orforglipron"[Title/Abstract] OR "LY3502970"[Title/Abstract]) AND (review[Title] OR "narrative review"[Title/Abstract] OR "systematic review"[Title/Abstract] OR "meta-analysis"[Publication Type] OR "network meta-analysis"[Title/Abstract])	Publication date: January 1, 2020 to April 30, 2026; Language: English; Article types: Review, Systematic Review, Meta-Analysis	Secondary literature was used primarily for contextualization and not as a substitute for primary evidence
Embase	Structural biology and receptor pharmacology	To expand retrieval of pharmacology, medicinal chemistry, and drug-development literature beyond MEDLINE indexing	(orforglipron:ti,ab,kw OR LY3502970:ti,ab,kw OR Foundayo:ti,ab,kw) AND ("GLP-1 receptor":ti,ab,kw OR "glucagon-like peptide-1 receptor":ti,ab,kw OR GLP1R:ti,ab,kw) AND (structure:ti,ab,kw OR "structural basis":ti,ab,kw OR "binding mode":ti,ab,kw OR "small molecule":ti,ab,kw OR nonpeptide:ti,ab,kw OR "biased agonism":ti,ab,kw OR "G protein-biased":ti,ab,kw OR "medicinal chemistry":ti,ab,kw OR "receptor pharmacology":ti,ab,kw)	Year: 2020–2026; Language: English; no Humans filter; Document types: article, article in press	Embase was used to improve sensitivity for pharmacologic and translational evidence
Embase	Comparative clinical evidence	To capture active-comparator studies and clinically oriented evidence not always retrieved in PubMed alone	(orforglipron:ti,ab,kw OR LY3502970:ti,ab,kw OR Foundayo:ti,ab,kw) AND (semaglutide:ti,ab,kw OR "oral semaglutide":ti,ab,kw OR dulaglutide:ti,ab,kw OR liraglutide:ti,ab,kw OR exenatide:ti,ab,kw OR lixisenatide:ti,ab,kw OR tirzepatide:ti,ab,kw) AND (versus:ti,ab,kw OR comparison:ti,ab,kw OR comparative:ti,ab,kw OR "head-to-head":ti,ab,kw OR superior:ti,ab,kw OR "non-inferiority":ti,ab,kw OR "indirect comparison":ti,ab,kw OR "network meta-analysis":ti,ab,kw)	Year: 2020–2026; Language: English; Humans; Document type: article; Study type: clinical trial, randomized controlled trial, comparative study	Particularly useful for strengthening retrieval of comparative and pharmacotherapy studies
Scopus	Broad replication and citation expansion	To replicate core searches and support citation tracking across biomedical and interdisciplinary literature	TITLE-ABS-KEY ( orforglipron OR LY3502970 OR Foundayo ) AND TITLE-ABS-KEY ( "GLP-1 receptor" OR "glucagon-like peptide-1 receptor" OR GLP1R OR semaglutide OR dulaglutide OR comparison OR "head-to-head" OR "small molecule" OR nonpeptide OR "structural basis" OR "biased agonism" )	Years: 2020–2026; Language: English; Document types: Article and Review	Scopus was used mainly for citation expansion and cross-validation of database retrieval
Web of Science Core Collection	Broad replication and citation expansion	To cross-check retrieval and perform forward/backward citation tracking	TS=(orforglipron OR LY3502970 OR Foundayo) AND TS=("GLP-1 receptor" OR "glucagon-like peptide-1 receptor" OR GLP1R OR semaglutide OR dulaglutide OR comparison OR "head-to-head" OR "small molecule" OR nonpeptide OR "structural basis" OR "biased agonism")	Timespan: 2020–2026; Language: English; Document types: Article and Review	Web of Science was used to complement citation tracking and ensure broader literature coverage
ClinicalTrials.gov	Trial identification and verification	To identify registered completed, ongoing, or recently reported interventional studies involving orforglipron	orforglipron	Study type: Interventional; Conditions: Type 2 diabetes mellitus, obesity; Status: Completed, Active not recruiting, Recruiting; Sponsor: Eli Lilly and Company	Used to verify trial registration, comparator design, and development-stage context
FDA / DailyMed / official U.S. prescribing information	Regulatory and practical prescribing comparison	To compare official administration instructions, food requirements, oral dosing constraints, and clinically relevant label information	FOUNDAYO prescribing information; RYBELSUS prescribing information	Most recent official U.S. label version only; no secondary summaries or commercial websites	Regulatory documents were used exclusively for official prescribing and administration claims
